# Effects of N-Alkanol Adsorption on Bubble Acceleration and Local Velocities in Solutions of the Homologous Series from Ethanol to N-Decanol

**DOI:** 10.3390/ma16052125

**Published:** 2023-03-06

**Authors:** Marcel Krzan, Pradipta Chattopadhyay, Sandra Orvalho, Maria Zednikova

**Affiliations:** 1 Jerzy Haber Institute of Catalysis and Surface Chemistry, Polish Academy of Sciences, 30-239 Krakow, Poland; 2 Department of Chemical Engineering, BITS-Pilani, Pilani PIN-333031, Rajasthan, India; 3 Institute of Chemical Process Fundamentals of the CAS, Rozvojová 1, 165 00 Prague, Czech Republic

**Keywords:** bubble, alkanols, adsorption coverage, acceleration, bubble velocity, fluidity of interface, adsorption kinetics, dynamic structure of adsorption layer

## Abstract

The influence of n-alkanol (C2–C10) water solutions on bubble motion was studied in a wide range of concentrations. Initial bubble acceleration, as well as local, maximal and terminal velocities during motion were studied as a function of motion time. Generally, two types of velocity profiles were observed. For low surface-active alkanols (C2–C4), bubble acceleration and terminal velocities diminished with the increase in solution concentration and adsorption coverage. No maximum velocities were distinguished. The situation is much more complicated for higher surface-active alkanols (C5–C10). In low and medium solution concentrations, bubbles detached from the capillary with acceleration comparable to gravitational acceleration, and profiles of the local velocities showed maxima. The terminal velocity of bubbles decreased with increasing adsorption coverage. The heights and widths of the maximum diminished with increasing solution concentration. Much lower initial acceleration values and no maxima presence were observed in the case of the highest n-alkanol concentrations (C5–C10). Nevertheless, in these solutions, the observed terminal velocities were significantly higher than in the case of bubbles moving in solutions of lower concentration (C2–C4). The observed differences were explained by different states of the adsorption layer in the studied solutions, leading to varying degrees of immobilization of the bubble interface, which generates other hydrodynamic conditions of bubble motion.

## 1. Introduction

Bubble motion can be found in the natural environment and industrial applications, such as mineral processing and material transportation, energy production, cooling and cleaning systems, chemical engineering or environmental protection processes [1,2,3,4]. In the case of foaming processes, the adsorption of surface-active substances on the bubble interface during its formation and the subsequent dynamic adsorption and desorption processes affect the size and stability of the generated foam [1,5,6]. Similarly, in the case of emulsion formation, the dynamics of surfactant adsorption on the drop interface have a critical impact on the stability of the obtained dispersed system [7,8].

The motion of rising bubbles is a complex problem since it involves interaction between buoyancy force (added mass impact) and drag and lift forces (state of the bubble interface and behavior of the liquid) [9,10,11,12,13]. In water devoid of surfactants, the bubble surface is fully mobile; therefore, the local bubble velocity is higher than that of a solid sphere of identical diameter and density. On the contrary, when bubbles rise in surface-active agent solutions, an uneven distribution of surfactant molecules over the bubble surface is induced due to a viscous drag exerted by the fluid moving around the bubble interface. As a result, adsorption coverage is lowered on the upstream part, while an accumulation of adsorbed molecules of surface-active substance takes place on the rear part of the bubble. This surface concentration gradient can reduce interfacial mobility and diminish the bubble’s velocity [14]. The steady-state conditions are already established at this stage, and a dynamic balance exists between all forces acting on the rising bubble.

However, despite the rather extensive data, many significant problems are still described only at a limited level. Moreover, the experimental data are often unclear or in disagreement with each other. Most of the existing experimental data describe the impact of high surface-active compounds on the terminal velocity of bubbles. In the literature, only the last stage of bubble motion has been described, when bubble moves at terminal velocity. For example, in the case of bubbles with diameter within the range of 1–2 mm rising in clean (distilled or filtered) water, values of the terminal velocity between 23 and 37 cm/s have been published [5,9,10,11,12,13,14,15,16,17,18]. For tap water, values varying between 14 and 38 cm/s have been reported [15,19,20]. Lower values of terminal velocity are undoubtedly related to surface-active contaminations present in tap water. For solutions of high surface-active compounds, values of terminal velocities of 14.5–17 cm/s have been obtained [19,21,22,23,24]. A recently published review paper [25], successfully correlated the terminal velocity of bubbles rising in surfactant solutions with the surfactant concentration for a wide set of surface-active molecules including n-alkanol solutions (C3-C9).

Complete profiles of local velocity variations, the degree of the bubble shape variations and time needed to establish a steady-state velocity as a function of solution concentration and type of surfactant are rarely presented in the literature on this subject [9,12]. However, even in cases in which a complete profile of bubble motion is described, only a limited number of results have been reported. This is because the literature presents only the most known and used high surface-active substances, such as sodium n-dodecyl sulfate [10,26,27], n-cetyltrimethylammonium bromide [11,28] or long-chained alkanols [9,11,28,29,30] and Tween [28]. To the best of our knowledge, no experimental verification of bubble motion parameters in a whole homologous series of any surface-active substance exists. Detailed description of the instantaneous velocity of bubbles in solutions of compounds with low and medium surface activity is also missing.

In this paper, we present the determination of initial acceleration, profiles of the local velocities and values of maximum and terminal velocities of bubbles rising in solutions of a homologous series of n-alkanols (C2–C10). Ethanol and n-propanol have low surface activity and are characterized by rapid adsorption kinetics [17,31,32,33,34,35]. On the other hand, n-octanol, n-nonanol and n-decanol have high surface activity, but their adsorption kinetics are so slow that the detached bubbles have minimal surface coverage [35,36,37].

We present the results for bubbles ranging from 1.3 to 1.5 mm, for which the Reynolds numbers vary from 600 in water to ca. 200 in contaminated solutions. We also evaluated the influence of solution concentration and n-alkanol chain length on a time scale establishing a steady-state and an uneven distribution of the surfactant molecules over the bubble surface for each studied n-alkanol. Finally, the analysis explains how the interfacial bubble immobilization effect depends on the type of adsorbed surfactant, compound surface activity and adsorption time.

## 2. Materials and Methods

Alkanols studied in this paper were purchased from Sigma-Aldrich in the highest available purity (at least 99.5%) and were used as delivered. All experiments were carried out at a room temperature of 22 ± 1 °C using Merck Millipore Direct-Q3 (Merck KGaA, Darmstadt, Germany) ultrapure water for solution preparation (conductivity: 0.05 μS/cm; resistivity: 18.2 MΩ cm; surface tension: 72.4 mN m^−1^ at 22 °C). A general cleaning procedure was applied to all the used glassware: (i) cleaning using a household detergent and rinsing with hot tap water; (ii) stored overnight (minimum of 10 h) in a container filled with solution of Mucasol universal detergent purchased from Aldrich (high pH); (iii) rinsing again with hot tap water and cleaning with a mixture of chromic acid and concentrated sulfuric acid; (iv) rinsing with twice-distilled water and with Milli-Q ultrapure water (conductivity: 0.05 μS/cm).

The basis of the setup used for precise determination of the local velocity profiles of bubbles detaching from the capillary and travelling through the liquid was previously described elsewhere [9,10,11,12,27]. It consists of a long (50 cm high), square (4 × 4 cm cross-section) glass column filled with ca 750 mL of solution. Individual bubbles were formed by the pinch-off bubble detaching method at the bottom of the column using a peristaltic pump connected to the capillary (inner diameter: 0.075 mm). The bubble expansion time (time available for surfactant adsorption over expanding gas/liquid interface) was always ca. 1.6 ± 0.2 s, while the delay before the next bubble was longer than 10 s. Stroboscopic light was used to obtain a few (3–6) different positions of the single rising bubble in one still movie frame. A Moticam digital USB camera equipped with a Nikkor 60 Macro lens recorded the bubble motion at various distances from the capillary orifice. The bubble local velocities were then automatically evaluated using our special macro of ImageJ digital analysis freeware. The local velocities at a given distance from the capillary were calculated using the coordinates of the subsequent positions of the bubble center of mass and the time intervals between the stroboscopic lamp flashes [9,10,11,12,27].

To determine the initial acceleration of the detached bubbles in the proximity of the capillary (up to ca. 20 mm from the bubble detachment point), we used the data collected by image analysis of the images captured by a Macrovis high-speed camera at acquisition frequency 1000 Hz. The acceleration ratio was evaluated using another particular macro of ImageJ digital analysis freeware. However, the general method of bubble motion evaluation was the same as in local bubble velocity analysis [38]. The bubble expansion time was not constant in some experiments, in order to investigate the effect of the surface coverage at the detachment on the initial acceleration.

## 3. Results

### 3.1. Local Velocities

**Figure 1:**
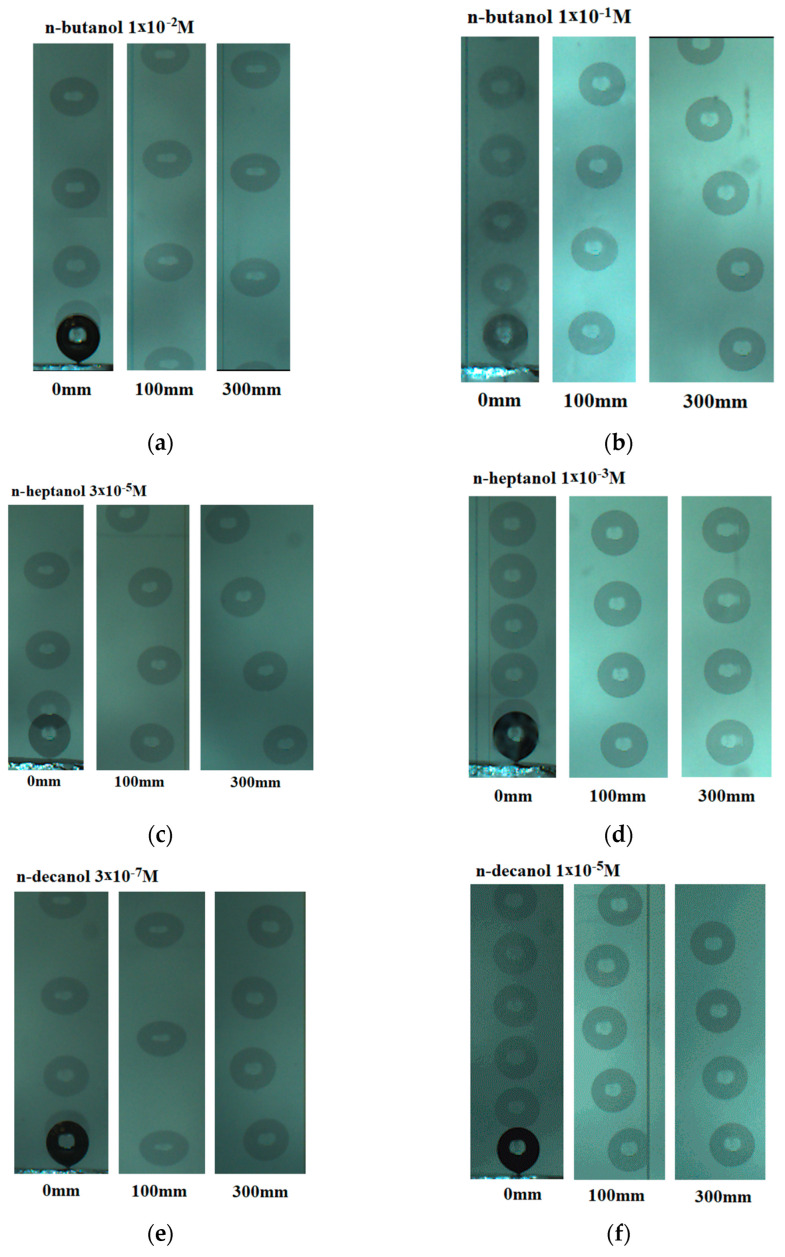
Images of bubbles rising in various n-alkanol solutions. (**a**) 1 × 10^−2^ M n-butanol; bubble diameter: 1.48 ± 0.01 mm; all experiments were recorded with a flash frequency of 100 Hz (0.01 s interval between images of the rising bubble). (**b**) 1 × 10^−1^ M n-butanol; bubble diameter: 1.37 ± 0.01 mm; experiments close to the capillary (0 mm) were recorded with a flash frequency of 100 Hz, and other experiments (100 and 300 mm from the capillary) were recorded with a flash frequency of 75 Hz (0.13 s interval between images of the bubble). (**c**) 3 × 10^−5^ M n-heptanol; bubble diameter: 1.45 ± 0.02 mm; all experiments were recorded with a flash frequency of 100 Hz. (**d**) 1 × 10^−3^ M n-heptanol; bubble diameter: 1.42 ± 0.02 mm; experiments close to the capillary (0 mm) were recorded with a flash frequency of 100 Hz, and other experiments (100 and 300 mm from the capillary) with a flash frequency of 75 Hz. (**e**) 3 × 10^−7^ M n-decanol; bubble diameter: 1.44 ± 0.02 mm; all experiments were recorded with a flash frequency of 100 Hz. (**f**) 1 × 10^−5^ M n-decanol; bubble diameter: 1.43 ± 0.02 mm; experiments close to the capillary (0 mm) were recorded with a flash frequency of 100 Hz, and other experiments (100 and 300 mm from the capillary) were recorded with a flash frequency of 75 Hz.

Figure 1 presents images of bubbles rising in n-butanol solutions of 1 × 10^−2^ M (Figure 1a) and 1 × 10^−1^ M (Figure 1b). In the case of the images in Figure 1b, a significant slowdown of the stroboscopic lamp rate (from 100 to 75 Hz) was required to capture the slow buoyant bubbles. The slower bubble motion in 1 × 10^−1^ M solution was related to the much more significant effect of the high concentration of n-butanol on the movement of the bubble. At a concentration of 1 × 10^−2^ M, the bubble moves in a straight line and has a spheroidal (ellipsoidal) shape. On the contrary, at a concentration of 1 × 10^−1^ M, a significantly smaller bubble is generated, which moves in a spherical (not deformed) form, and its movement is considerably slower. Moreover, the bubble trajectory shifts from a straight line close to the capillary to a helical (spiral) trajectory at a distance of 300 mm.

**Figure 2:**
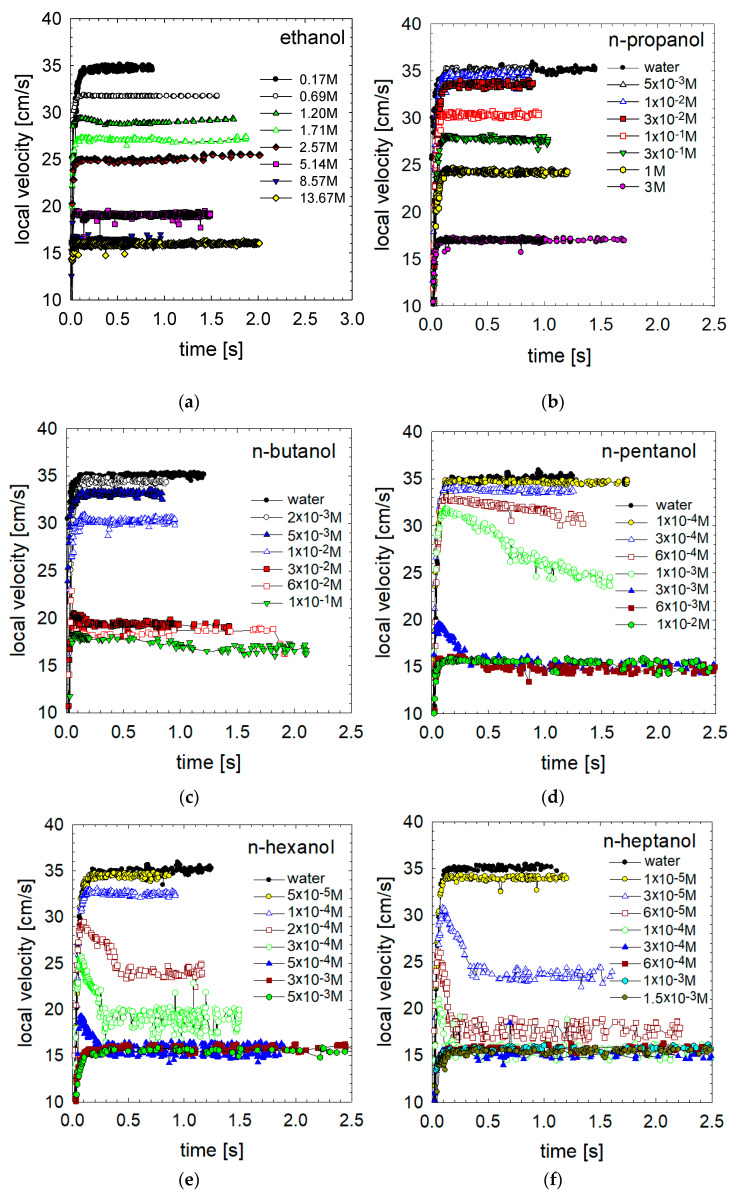

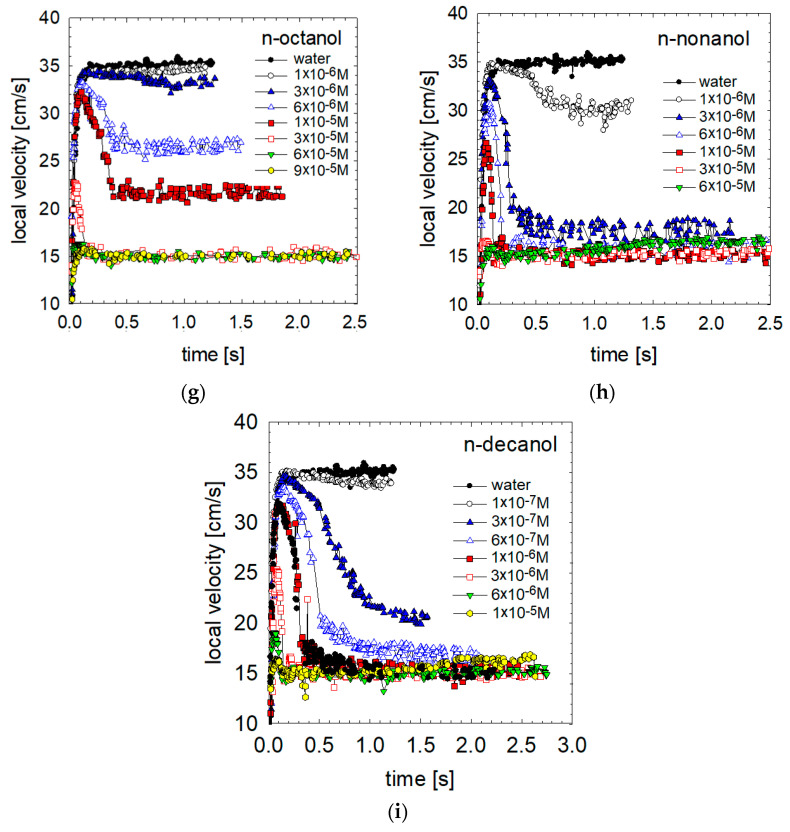
Bubble local velocity as a function of time in solutions: (**a**) ethanol, (**b**) n-propanol, (**c**) n-butanol, (**d**) n-pentanol, (**e**) n-hexanol, (**f**) n-heptanol, (**g**) n-octanol, (**h**) n-nonanol and (**i**) n-decanol. (t = 0—time of the bubble detachment from the capillary).

Figure 2 presents quantitative data on the effect of n-alkanol (C2–C10) concentration on profiles of the bubble’s local velocity. Bubbles accelerated rapidly immediately after departing from the capillary.

In solutions of low surface-active compounds (ethanol, n-propanol or n-butanol; Figure 2a, Figure 2b and Figure 2c, respectively) with relatively high concentrations and fast adsorption kinetics, the bubble detached with a considerably uniform surface coverage. However, the bubble acceleration was relatively high, and only two motion stages were observed: acceleration and terminal velocity. Rapid adsorption/desorption kinetics are the most probable factor preventing an establishment of a steady-state uneven distribution of low surface-active n-alkanol molecules across the interface of the rising bubbles due to the viscous drag acting on the bubble surface. The lack of a deceleration stage confirms that assumption. Together with Dukhin and Frumkin-Levich’s theories [5,6,14], the deceleration stage occurs when the amount of adsorbed surfactant molecules is sufficient to attain a rear stagnant cap configuration. Therefore, we can conclude that in such cases, the adsorption and desorption fluxes are almost perfectly balanced, and no significant accumulation of alkanol molecules occurs on the bottom pole of the bubble.

Figure 1c,d show bubble movement in n-heptanol solutions with concentrations of 3 × 10^−5^ M and 1 × 10^−3^ M. As shown in Figure 1c, in the case of a concentration of 3 × 10^–5^ M, the bubble moves in a helical (spiral) trajectory, which is related to the maximum velocity and deceleration stage observed at this concentration in Figure 2f. On the contrary, in the 1 × 10^–3^ M solution, the movement trajectory remains linear because the bubble moves much slower from the moment of detachment from the capillary, and the stages of high acceleration, maximum velocity and deceleration are not observed here (Figure 2f).

As seen in Figure 2d–f, in low concentrations of medium surface-active alkanols (with hydrocarbon chain 5 ≤ C ≤ 7), the accelerating peak and a deceleration stage occur before reaching terminal velocity. The declination of the deceleration curve also varies depending on the n-alkanol chain length. In the case of n-pentanol or n-hexanol, a gentle, slow decrease in the bubble velocity is observed. The terminal velocity of the bubble can considerably exceed 15 cm s^−1^. The maximum of the local velocity dependences is probably an indication that at these lowest n-alkanol concentrations, the departing bubble is in a period of induction of the surface tension gradients. It can be assumed that for alkanols with hydrocarbon chain 5 ≤ C ≤ 7, the ratio between adsorption and desorption fluxes is already unbalanced and that the desorption rates are lower than the adsorption rate. As a result, a stagnant cap is formed as described by Frumkin and Levich [5,6,14]. The accumulation of surfactant molecules at the bottom part of the bubble leads to a much higher Marangoni effect than in the case of low surface-active alkanols. Consequently, the local bubble velocity profiles peaked, and the deceleration stage occurred as predicted by the theories proposed in [5,6,14]. We can distinguish three phases of bubble motion: (i) initial rapid acceleration until reaching a maximum velocity, (ii) a monotonic decrease in the velocity and (iii) a constant value of the terminal velocity. The maximum heights and widths varied (diminished) with the solution concentration and surface activity. The observed diminishment of the bubble terminal velocity is strictly related to the solution concentration and the initial degree of adsorption on the bubble interface during its detachment.

Figure 1e,f show the bubble movement in n-decanol solutions with concentrations of 3 × 10^−7^ M and 1 × 10^−5^ M. At both concentrations, we observed bubbles rising in a helical (spiral) path. Local velocity profiles in n-decanol (Figure 2i) confirm our hypothesis that helical motion is related to the presence of velocity peak and subsequent deceleration stage.

In the highest surface-active compounds, n-nonanol or n-decanol, a sharp peak and rapid velocity deceleration occurred after the maximum (Figure 2g–i). The deceleration stages almost always finished with a terminal velocity value equal to 15 cm s^−1^, independent of the solution concentration and the surfactant.

Significant differences in the profiles of local bubble velocities in various n-alkanols can be explained only by the different adsorption processes on the bubble interface in different n-alkanols caused by their unique surface-active properties. Let us analyze n-pentanol and n-hexanol local velocity profiles (Figure 2d,e). Here, we can distinguish various types of “deceleration” curves. At the lowest solution concentrations, i.e. 1 × 10^−4^ M and 3 × 10^−4^ M n-pentanol and 5 × 10^−5^ M n-hexanol, the bubble accelerated and achieved maximum velocity, after which it rose with the same terminal velocity. It can be concluded that the bubble behaved as in the case of low surface-active alkanols. Due to low concentrations, the adsorption and desorption fluxes are balanced and no significant accumulation of the surfactant molecules occurs on the bottom part of the bubble. As a result, no significant Marangoni effect occurs, and no deceleration occurs in the limited distance of motion of 50 cm.

The local velocity profiles in 6 × 10^−4^ M and 1 × 10^−3^ M of n-pentanol or 1 × 10^−4^ M n-hexanol reflect another situation. Here, after acceleration and reaching the velocity peak, a slight deceleration of the bubble motion was observed. Due to the limited column size, the velocity reduction did not reach a constant terminal value. Therefore, we could not estimate a bubble terminal velocity, which could be achieved over a longer distance of motion. We only know that it will be between the last recorded value of local velocity and ca. 15 ± 1 cm/s (the minimum value of bubble velocity in contaminated solutions of surface-active substances [9,10,11,26]). With a further increase in concentration to 2 × 10^−4^ M and 3 × 10^−4^ M of n-hexanol, a reduction in the bubble velocity was observed, and a constant terminal velocity was reached, which is still higher than 15 cm/s. Further increase of the concentration led to a significant reduction in the initial bubble acceleration, and the maximum velocity stage disappeared. After a short acceleration stage, the bubble rose with a terminal velocity of about 15 cm/s.

### 3.2. Acceleration

**Figure 3:**
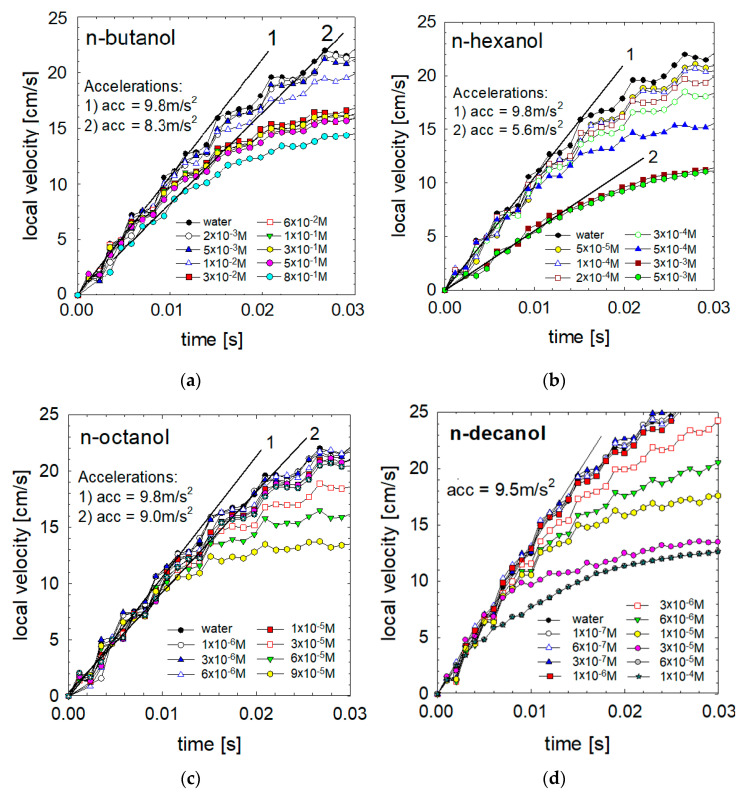
Detailed velocity profiles (for acceleration stage of motion) as a function of (**a**) n-butanol, (**b**) n-hexanol, (**c**) n-octanol and (**d**) n-decanol solutions of different concentrations. Slopes of lines 1 and 2 correspond to the initial acceleration of few chosen cases.

To verify the effect of the initial adsorption on the movement of the bubble, we performed acceleration measurements using a high-speed camera with image acquisition rate of 1040 Hz. As seen in Figure 3, at low and medium concentrations of the studied alkanols (n-butanol, n-hexanol, n-octanol and n-decanol), initial bubble acceleration was almost unaffected by the presence of surface-active compounds. The bubble rose with almost gravitational acceleration (ca. 9.30–9.80 m/s^2^), as in pure water. Only at high concentrations of some surface-active agents, such as 5 × 10^−3^ M n-hexanol, bubbles rose after detachment with acceleration ca. 5.6 m/s^2^. An interesting situation also occurred in the case of the highest concentration of n-decanol. The bubble detached from the capillary and moved for the first 1/300 s with an acceleration of 950 cm/s^2^. Only later, its acceleration rapidly decreased to 6.0 m/s^2^.

**Figure 4:**
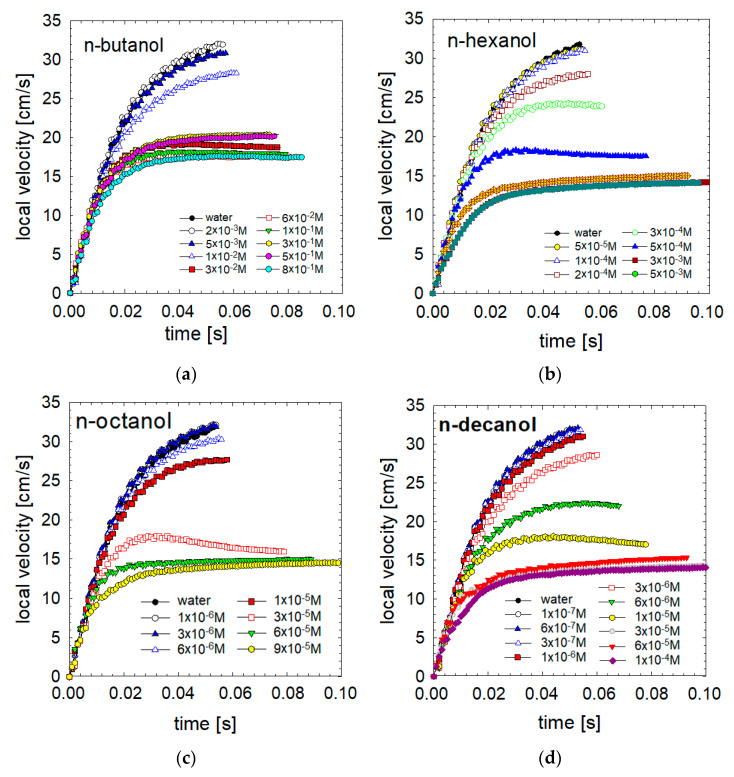
Detailed velocity profiles (for acceleration stage of motion) as a function of solution concentration in (**a**) n-butanol, (**b**) n-hexanol, (**c**) n-octanol and (**d**) n-decanol solutions of different concentrations.

Figure 4 presents the bubble velocity profile over an extended time, up to 0.1 s. This period was sufficient in all cases for the generation of a dynamic adsorption layer and to noticeably reduce the initial acceleration rate. These profiles prove that the local velocities were determined by adsorption and hydrodynamic processes occurring at and around the bubble interface. In the case of low and medium surfactant concentrations, the bubble’s initial acceleration decreased to practically zero within 0.06 s, after which the bubble reached its maximum speed and began the deceleration stage. On the contrary, in the case of high surfactant concentrations, this time was more than sufficient to reach the terminal velocity of motion.

### 3.3. Adsorption Time

**Figure 5:**
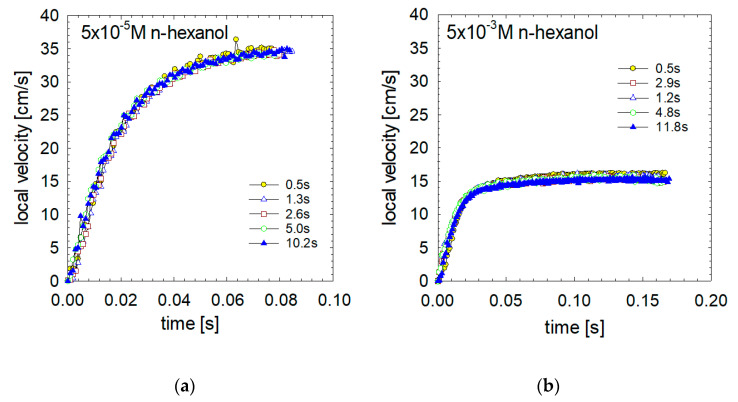
Detailed velocity profiles (for acceleration stage of motion) in n-hexanol solutions (**a**) 5 × 10^−5^ M and (**b**) 5 × 10^−3^ M as a function of the detachment time.

**Figure 6:**
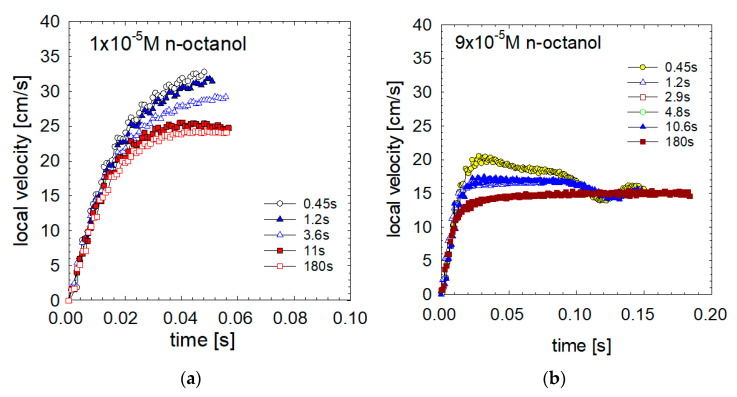
Detailed velocity profiles for acceleration stage of motion) in (**a**) 1 × 10^−5^ M and (**b**) 9 × 10^−5^ M n-octanol solution as a function the detachment time.

We studied the effect of adsorption time on bubble acceleration in solutions of n-hexanol (Figure 5) and n-octanol (Figure 6). Two time instants were considered: first, the detachment time or the adsorption time, corresponding to the time it takes a bubble to grow before it detaches from the capillary tip, and second, when the bubble surface reached equilibrium state. To these two instants correspond different degrees of adsorption: θ_detach._ and θ_equil._, respectively. The degrees of adsorption were calculated using a methodology developed by Warszynski et al. [39] and Jachimska et al. [40]. The Warszynski model [39,40] assumes that (i) the bubble grows uniformly, (ii) surfactant molecules are transferred to the interface by the convective-diffusion mechanism and (iii) adsorption kinetics are described by the Frumkin–Hinshelwood model (which, in equilibrium, is consistent with the Frumkin adsorption isotherm). In our previous work, we found agreement between the model and the experimental data [9,10,11,12].

As seen in Figure 5, in the case of n-hexanol, adsorption time had no impact on bubble acceleration and further motion, independent of the n-hexanol concentration. Furthermore, profiles of the local bubble velocities in 5 × 10^−5^ M (Figure 5a) (Figure 5b) of n-hexanol are precisely the same independent of the different adsorption period (adsorption times varied between 0.5 and 11 s). The same is observed for the concentration of the solution 5 × 10^−3^ M (Figure 5b).

In n-octanol (Figure 6), the detachment time (and achieved degree of adsorption coverage) strongly affected the bubble motion. In the case of 1 × 10^−5^ M n-octanol solutions (Figure 6a), bubbles detached with a initial acceleration of about 9.3 m/s^2^, identical to the acceleration in n-hexanol solutions, that is independent of the detachment time and concentration of n-hexanol. However, contrary to the n-hexanol cases, after ca 0.01–0.02 s of motion, the initial acceleration faded, and the deceleration follow closely related the adsorption time (short detachment time—small deceleration, long detachment time—big deceleration). 

The initial acceleration, for the 9 × 10^−5^ M n-octanol solution (Figure 6b), was also 9.3 m/s^2^. However, depending on the detachment time, we observed two types of evolution: either (i) acceleration, a maximum speed of 20 cm/s and slowing down to the limiting velocity in 0.1 s, or (ii), acceleration–deceleration and transition to the terminal speed stage without the observed maximum velocity, as observed for detachment time of 180 s.

These results prove that in the case of n-hexanol, the adsorption kinetics are so fast that even a minimum time for adsorption (small detachment time) is more than enough to obtain the equilibrium adsorption state. As a result, the bubble detaches with uniform and equilibrium adsorption, and the degree of adsorption depends only on the bubble size and solution concentration. In contrast, in n-octanol solutions, the much slower adsorption kinetics lead to various adsorption coverages over the interface of the detached bubble, depending on the applied detachment times.

Table 1 and Table 2 present the numerical calculations of the adsorption coverages at detachment and at equilibrium for n-hexanol and n-octanol solutions. As seen in Table 1, the n-hexanol degree of adsorption on the detached bubble depends only on the solution concentration and is practically independent of the adsorption time. Even an interval as short as 0.5 s is enough to obtain almost equilibrium adsorption coverages (90% of equilibrium). In contrast, in all of the studied solutions of n-octanol, the equilibrium degree of adsorption was not reached. Even a long duration of bubble formation of 180 s was not enough to reach the equilibrium degree of adsorption. These results are related to the higher surface activity and slower kinetics of adsorption of n-octanol. Generally, we can conclude that in solutions of surfactants, which have low surface activity and fast adsorption/desorption kinetics such as n-hexanol (or ethanol—n-pentanol), the bubble interface upon detachment from the capillary orifice had the equilibrium adsorption coverage. Moreover, rapid diffusion means fast transport to and from the interface at every motion stage. Thus, any deviations from equilibrium are readily compensated for by the diffusion and adsorption processes and supported by the hydrodynamic transport of the surfactant to the surface of the bubble.

On the contrary, in the case of n-octanol ( the solution of high surface activity and slow adsorption kinetics), there is a non-equilibrium adsorption coverage at the interfaces of the detaching bubbles does not reach the equilibrium, even after a long bubble formation period (180 s). The adsorbed molecules of n-octanol are uniformly distributed until the time of detachment, but bubble motion induces the dynamic structure of the adsorption layer. As the adsorption kinetics is much slower, convection over the bubble surface can prevail. Therefore, the adsorbed molecules are immediately shifted through the interface and accumulate on the bottom part of the bubble. Consequently, a strong surface tension gradient is created, and bubble interface mobility is hindered at low adsorption coverage in the case of high-surface-activity solutes.

**Figure 7:**
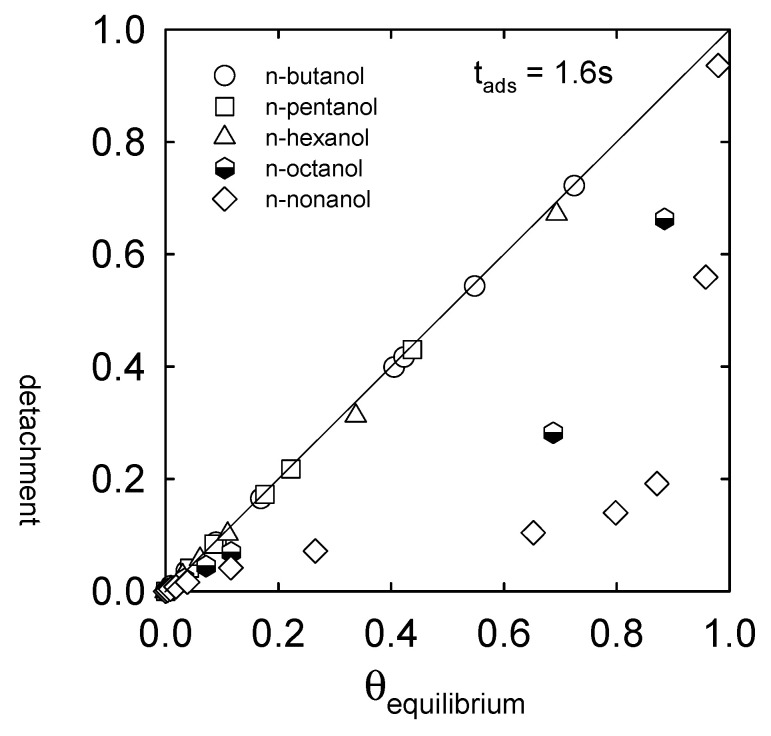
The adsorption coverage over the surface of the departing bubble (t_ads_ = 1.6 s) as a function of the equilibrium adsorption coverage.

Figure 7 presents the adsorption coverages over the surface of the departing bubbles (t_ads_ = 1.6 s) as a function of the equilibrium adsorption coverage. If the detaching bubbles have equilibrium adsorption coverages, the data should be on line 1:1. Points below line 1:1 indicate non-equilibrium adsorption coverages. As seen in low and moderate surface-active agent solutions (up to n-hexanol), the bubble detaches under equilibrium adsorption conditions. On the contrary, in higher surface-active agents, the bubbles depart with non-equilibrium adsorption coverages.

### 3.4. Terminal Velocities

**Figure 8:**
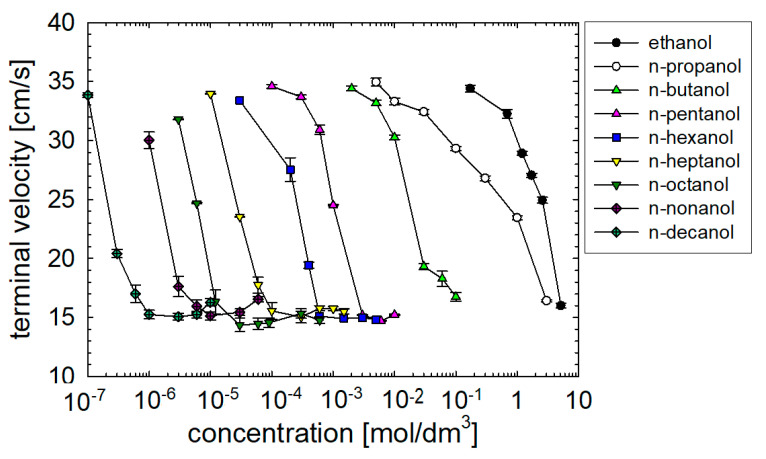
Terminal velocity of bubbles in n-alkanol solutions as function of their concentration. All bubbles with detachment time = 1.6 s.

Dependencies of the bubble terminal velocities on n-alkanol concentration are presented in Figure 8. The terminal velocities correspond to the average value of local velocities at a distance of 350–500 mm from the capillary. In most cases, so far away from the capillary, the bubbles rise with a constant velocity. The terminal velocity of a 1.5 mm bubbles rising in distilled water is 34.8 ± 0.3 cm s^−1^ [9,10,11,12,23,24,26,27,28,29,41]. As seen in Figure 7, the trend is the same in all cases. Increasing the concentration of n-alkanol, the terminal velocity falls to a minimum, common to all the studied n-alkanols, of 15 ± 1 cm s^−1^. The minimum terminal velocity values probably correspond to a maximum surface tension gradient between the bubble front and rear poles. Such conditions are function of the surface-active molecule properties and the bubble size. The critical concentration necessary for the reduction of the terminal velocity decreases with the increase of the chain length. 

It can be assumed that this critical concentration is function of bubble size, alkanol surface activity, adsorption kinetics, initial adsorption time (detachment time) and time the bubble has been in contact with the liquid during its rise. Alkanol surface activity and adsorption kinetics depend on alkyl chain length. Bubble size dependents on bubble formation conditions (capillary size and surfactant surface activity [9,10,11,12,23,24,26,27,28,29,41]). Further increase of concentration above the critical concentration is passed leads to a small increase of terminal velocity value in long-chain alkanols. For example, in n-decanol, the 1 × 10^−6^ M concentration bubbles rise with a terminal velocity of 15.3 ± 0.3 cm s^-1^ (at a distance of 500 mm from the capillary tip). While, for a ten times higher concentration of 1 × 10^−5^ M, a velocity of 16.3 ± 0.3 cm s^−1^ is observed. Higher terminal velocity in a higher concentration of the studied compound is observed in all the long-chain n-alkanol (C ≥ 8). This may indicate that the steady-state conditions obtained by the bubble in the terminal velocity stage of motion depended on the concentrations of surfactants.

In diluted and moderate solutions of high surface-active substances, the bubble detaches from the capillary almost free from surfactant and completely mobile interface (1.6 s of bubble growth is not enough to obtain an adsorption degree with noticeable effect on bubble terminal velocity). As a result, during the rise in these solutions, we observe initial high acceleration—almost the same as in pure water. Bubble motion deceleration occurs as a result of the ongoing adsorption of the surfactant molecules on the bubble during the motion, creating a dynamic adsorption layer. The generated Marangoni effect immobilizes the bubble interface and decelerates the rising bubble.

For high concentrations of high surface-active compounds, the initial adsorption degree of the surfactant during bubble detachment is already enough to reduce bubble acceleration immediately at the detachment from the capillary. As a result, the bubble accelerates less (ca. 30% slower), and no maximum is observed on the velocity profiles. Finally, we notice that the bubble rises with a terminal velocity higher than in the case of dilute or moderated concentrations. We believe that this indicates a much smaller Marangoni effect caused by lower gradient of surface tension difference between the bubble front and rear. In dilute or moderate solutions of high surface-active surfactants, the top part of the bubble is always free from surfactants, which are almost immediately transported due to the viscosity of the liquid on the bottom part. At high concentrations, the top pole is covered by some surfactant molecules, which are continuously, relatively rapidly and sufficiently transported from the solution to the top bubble interface due to hydrodynamic and adsorption processes.

### 3.5. Maximum and Terminal Velocities vs. Adsorption Coverages

**Figure 9:**
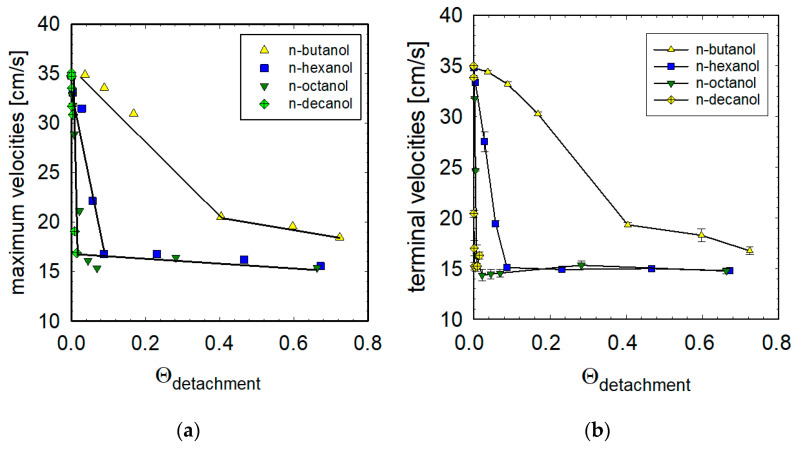
Maximum (**a**) and terminal (**b**) velocities as a function of the adsorption coverage upon detachment.

Figure 9 presents the relationship between adsorption coverages (time of detachment 1.6s) and the obtained maximum (Figure 9a) and terminal (Figure 9b) velocities. As seen, the terminal velocity values decrease rapidly with the adsorption coverage, but then start to be almost constant, above a definite coverage. These variations of the terminal velocity with adsorption coverage can be approximated, by two lines of very different slopes. As it was showed previously [9,10,11,12], the intersection of these two lines indicates value of the minimum adsorption coverage necessary for full immobilization of the bubble interface. The terminal velocity stages appear at the same degrees of adsorption as the maximum; however, the observed terminal velocities are slightly lower than corresponding maximum velocities.

We can see one significant difference between substances with low surface activity (i.e., n-butanol) and those with higher surface activity (n-hexanol, n-octanol and n-decanol). In the case of compounds with higher surface activity, the disappearance of the maximum velocity (Figure 9a) or achievement of the terminal velocity stage (Figure 9b) is indicated by a bubble motion with a speed of about 15 cm s^−1^. This is the classic situation described as fully developed dynamic adsorption layer, under which the bubble moves as with the fully immobilized interfacial surface [5,9,10,11,12,23,24,26,27,28,29,41].

In contrast, in the case of n-butanol, higher terminal velocities are possible, indicating that the dynamic adsorption layer is a result of a certain dynamic equilibrium between the adsorption processes at the bubble upper pole and desorption from its lower pole.

In addition, it is possible to determine the exact degree of adsorption for which the maximum disappears or terminal velocity stages appear. We can successively determine boundary surface coverages necessary for the formation of a dynamic adsorption layer, i.e., 40% for n-butanol, 10% for n-hexanol, 5% for n-octanol and less than 2% for n-decanol.

### 3.6. Mixtures of the High and Low Surface-Active Agent Compounds

**Figure 10:**
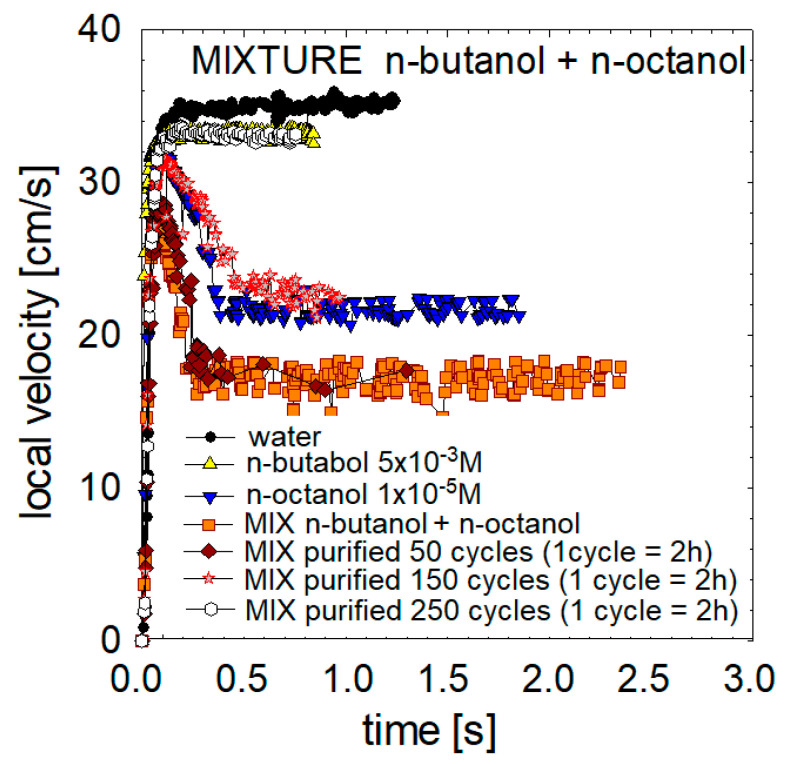
Profiles of bubble local velocities in a mixture of 5 × 10^−3^ M n-butanol and 1 × 10^−5^ M n-octanol.

In the last research stage, we decided to investigate how bubbles behave in mixtures of compounds with low and high surface activity. For this purpose, a mixture of butanol 5 × 10^−3^ M with n-octanol 1 × 10^−5^ M was prepared. The local velocity profiles of the mixture were compared with previously obtained data for 5 × 10^−3^ M butanol solutions with 1 × 10^−5^ M n-octanol (Figure 10). As shown in Figure 10, the bubbles in the mixture achieve lower accelerations, maximum and limiting velocities than in each component solution (n-octanol or n-butanol). Therefore, it can be assumed that there is a combination of surface interactions of both compounds, which coabsorb on the surface of the formed and rising bubble.

To verify this assumption, we carried out a partial purification of the mixture solution and controlled removing of the agent with higher surface activity and lower adsorption kinetics. For this purpose, we used the procedure and apparatus described in [42]. We conducted 50, 150 and 250 two-hour cycles of purification. In the obtained solutions, we carried out a standard measurement of the local velocity profiles of rising bubbles. The obtained results confirmed the achievement of higher accelerations, maximum and terminal velocities than in the initial mixture of 5 × 10^−3^ M butanol 1 × 10^−5^ M n-octanol. In the last test (after 250 cycles) we have got exactly the same profiles as for the pure 5 × 10^−3^ M n-butanol solutions. It indicates that all n-n-octanol was removed from the mixture. However, as seen from the profiles of mixture after 50 and 150 cycles, even trace amounts of n-octanol in the mixture have a critical effect on local velocities and bubble motion.

## 4. Discussion

Bubble acceleration, as well as local, maximal and terminal velocities, was found to be strongly dependent on the presence of n-alkanols. Even the most negligible surfactant content affects the parameters of the movement of bubbles. However, depending on the strength of the surface activity of the n-alkanol, we observed various changes in local velocity profiles.

Most of the existing experimental literature data describe the movement of bubbles into compounds with high surface activity. In the case of our research, these correspond to n-octanol, n-nonanol or n-decanol. In such solutions, at the low concentration, three stages of motion are observed: high initial acceleration (a ≈ 9.3 ms^−2^) until maximum velocity, deceleration and a terminal velocity stage. In high concentration solutions, the initial bubble acceleration is smaller (ca. 6 ms^−2^) and steady velocity state follows, without the maximum peak and deceleration stage in the velocity profile. The values of terminal velocity also depend on the concentration of alkanol, they decrease with concentration, till they reach a minimum value (15 cm s^−1^). At very concentrated solutions, the terminal velocity was of the order of 16.4 cm s^−1^. This increase, at very high concentrations can be explained, by the formation of a less effective dynamic adsorption layer in concentrated solutions because the bubble front might no longer completely free of surfactant molecules. Thus, the bubble rising in a very concentrated solution has a smaller surface tension gradient then a bubble rising in a solution with smaller concentration, corresponding to the observed minimum in terminal velocity.

The situation is similar in the case of moderate surface-active agent solutions (such as n-pentanol, n-hexanol or n-heptanol). Here, for the diluted solutions, the profiles of the bubble local velocities also exhibit a maximum in the local velocity profile. However, the reduction in the terminal velocity is not so strong. The bubble can rise here with a constant terminal velocity, exceeding 15–16 cm s^−1^.

In low surface-active compounds (ethanol, n-propanol or n-butanol) with relatively high concentrations and immediate adsorption kinetics, the bubble detached, with a considerably uniform surface coverage. Only two motion stages are observed: acceleration and terminal velocity, which depend on the solution concentration and can be much higher than the expected 15 cm s^−1^. The rapid adsorption/desorption kinetics are the most probable factor promoting the establishment of a steady-state distribution of low surface-active n-alkanol molecules across the interface of the rising bubbles, leading to the bubble movement also reaching steady state with constant velocity. It can be assumed that the desorption at the bottom of the bubble balances the adsorption process at the top of the bubble interface.

Values of the adsorption coverages assuring the disappearance of maximum velocity on the local velocity profiles were about 40% for n-butanol, 10% for n-hexanol, 5% for n-octanol and less than 2% for n-decanol, diminishing with increasing surface activity of the solution.

It was also shown that in the case of mixtures of compounds with low and high surface activity, the substances coadsorbed on the surface of the rising bubble. In such a situation, even minimal content of substances with high surface activity has a critical impact on the movement of the bubble and its local velocities.

## Figures and Tables

**Table 1:** Detaching and equilibrium adsorption coverages for n-hexanol solutions of 5 × 10^−5^ M and 5 × 10^−3^ M for various adsorption times.

Conc. (M)	Time (s)	θ_detach._ (%)	θ_equil._ (%)	Conc. (M)	Time (s)	θ_detach._ (%)	θ_equil._ (%)
5 × 10^−5^	0.5	0.66	0.74	5 × 10^−3^	0.5	65.2	69.2
5 × 10^−5^	1.3	0.69	0.74	5 × 10^−3^	1.2	66.7	69.2
5 × 10^−5^	2.6	0.71	0.74	5 × 10^−3^	2.9	67.6	69.2
5 × 10^−5^	5	0.71	0.74	5 × 10^−3^	4.8	68.0	69.2
5 × 10^−5^	10.2	0.72	0.74	5 × 10^−3^	11.8	68.4	69.2
5 × 10^−5^	180	0.73	0.74	5 × 10^−3^	180	69.0	69.2

**Table 2:** Detaching and equilibrium adsorption coverages for n-octanol solutions of 1 × 10^−5^ M and 9 × 10^−5^ M for various adsorption times.

Conc. (M)	Time (s)	θ_detach._ (%)	θ_equil._ (%)	Conc. (M)	Time (s)	θ_detach._ (%)	θ_equil._ (%)
1 × 10^−5^	0.45	0.55	1.1	9 × 10^−5^	0.45	5.1	11.6
1 × 10^−5^	1.2	0.68	1.1	9 × 10^−5^	1.2	6.5	11.6
1 × 10^−5^	3.6	0.81	1.1	9 × 10^−5^	2.9	7.7	11.6
1 × 10^−5^	11	0.91	1.1	9 × 10^−5^	4.8	8.3	11.6
1 × 10^−5^	180	1.03	1.1	9 × 10^−5^	180	9.2	11.6

## Data Availability

The data presented in this study are freely available from the authors upon reasonable request.
